# Acute Pancreatitis-Associated Thrombotic Venopathy

**DOI:** 10.7759/cureus.88624

**Published:** 2025-07-23

**Authors:** George S Zacharia, Navya Mandalapu, Saran Lal A Mokan Dasan, Sindhu K Gadde, Shivani Bansal, Sudha Bansal

**Affiliations:** 1 Internal Medicine, BronxCare Health System, Bronx, USA; 2 Medical Education, American University of the Caribbean School of Medicine, Cupecoy, SXM

**Keywords:** dvt, endothelial dysfunction, mesenteric vein thrombosis, pancreatitis, pulmonary embolism

## Abstract

Acute pancreatitis, a common cause of acute abdomen, is known to have a broad spectrum of local and systemic complications. Vascular complications of acute pancreatitis are vastly underreported. Our case, a 66-year-old male patient with alcohol-induced acute pancreatitis and concurrent severe ethanol withdrawal syndrome, who developed extensive thrombotic events early in the disease course, is a rare and intriguing presentation. The patient had concurrent thrombosis involving the distal superior mesenteric vein, the greater saphenous vein, and pulmonary embolism. This case highlights a multifocal and early onset of venous thrombosis complicating acute pancreatitis, an atypical but clinically significant presentation. The role of anticoagulation in patients with isolated splanchnic venous thrombosis in acute pancreatitis remains unclear; however, our patient was managed with low-molecular-weight heparin, owing to the presence of pulmonary embolism and extremity vein thrombosis. Further studies are needed to guide optimal management strategies in such complex presentations.

## Introduction

Acute pancreatitis ranks high among the causes of acute abdomen, with severity ranging from an uncomplicated inflammation limited to the pancreas to a complex disease with multiorgan dysfunction. The complications could be local, such as necrosis, fluid collections, or pseudocysts, or systemic, including acute kidney injury, acute respiratory distress syndrome, or systemic inflammatory response syndrome. Vascular complications, which are relatively rare in acute pancreatitis, are well-documented and can involve either the arterial or venous system. The inflammatory response associated with acute pancreatitis triggers a hypercoagulable state, which can lead to the development of venous thrombosis. Splanchnic venous thrombosis is reported in up to 50% of patients with necrotizing pancreatitis, while it is less frequent in those without necrosis, ranging from 1% to 17% [1-3]. Prospective studies have reported a deep venous thrombosis incidence of approximately 5% in patients admitted with acute pancreatitis, and have been linked with the severity and mortality of pancreatitis [4]. Venous thrombosis is frequently limited to a single venous territory, and simultaneous involvement of veins is relatively uncommon [1]. We here report a case of extensive thrombosis, including the superior mesenteric vein, the extremity veins, and pulmonary embolism complicating acute pancreatitis.

## Case presentation

A 66-year-old male patient presented with abdominal pain, nausea, vomiting of three days duration, followed by tremors of one day duration. He reported daily use of ethanol until the onset of gastrointestinal symptoms. Additionally, he reported a history of hypertension, hyperlipidemia, chronic obstructive pulmonary disease, and lumbosacral degenerative disc disease with sciatica. His medications included metformin, rosuvastatin, inhalational albuterol, and inhalational long-acting antimuscarinic, beta-agonist, steroid combination. At presentation, he was tachycardic, 129 beats per minute, hypertensive 149/101 mm of Hg, tremulous, anxious, and restless. He also reported intermittent auditory hallucinations but denied tactile or visual hallucinations. His estimated Clinical Institute Withdrawal Assessment for Alcohol, Revised (CIWA-Ar) score was 16 at arrival, suggesting severe ethanol withdrawal syndrome. Abdominal examination revealed tenderness in the upper abdomen, most severe in the epigastrium and left upper quadrant, with no rigidity or rebound tenderness.

**Figure 1:**
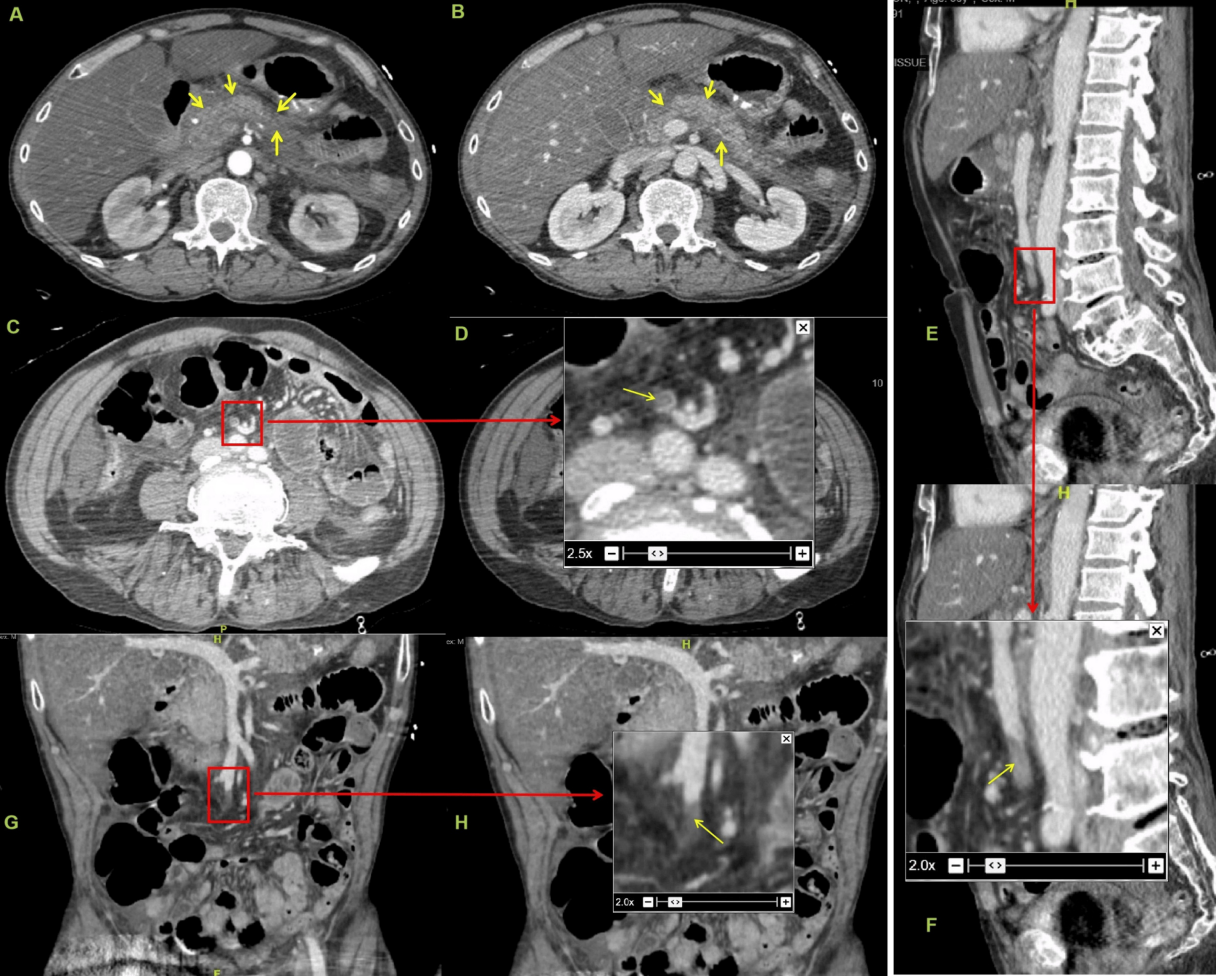
Contrast-enhanced computed tomography of abdomen Contrast-enhanced computed tomography of abdomen. (A & B) reveal bulky pancreas with indistinct borders and peripancreatic edema (yellow arrows). (C) Axial image with filling defect in the superior mesenteric vein (red box). (D) Magnified (2.5x) view of the superior mesenteric vein thrombus. Sagittal (E & F) and coronal (G & H) also demonstrated the superior mesenteric vein thrombus

The hemogram revealed leukocytosis, while the biochemical panel identified elevated lipase, transaminases, and hypokalemia (Table 1). Electrocardiogram reported sinus tachycardia, while a small left-sided pleural effusion was demonstrable in chest X-ray. A computed tomography (CT) with contrast of the abdomen and pelvis demonstrated features of acute pancreatitis (Balthazar grade C; CT severity index (CTSI) 2), hepatic steatosis, mild ascites, and a short segment thrombosis involving the distal superior mesenteric vein (Figure 1).

**Table 1:** An excerpt of laboratory work up at admission

Parameter	Results	Reference range
Hemoglobin	15.2	12-16 g/dL
Hematocrit	44.4%	42-51%
Mean corpuscular volume	110	80-96 fL
Leukocyte count	13	4.8-10.8 k/μL
Platelets	133	150-400 k/μL
Sodium	142	135-145 mEq/L
Potassium	2.7	3.5-5 mEq/L
Blood urea nitrogen	14	7-20 mg/dL
Creatinine	1.4	0.5-1.5 mg/dL
Calcium	8.4	8.5-10.5 mg/dL
Phosphorous	2.2	2.5-4.5 mg/dL
Bilirubin total	2.6	0.2-11 mg/dL
Bilirubin direct	1.1	0-0.3 mg/dL
Aspartate aminotransferase	138	9-36 U/L
Alanine aminotransferase	40	5-40 U/L
Alkaline phosphatase	100	43-160 U/L
Albumin	3.8	3.5-5.5 g/dL
Lipase	2362	<61 U/L
C-reactive protein	58	<5 mg/dL
Triglyceride	87	55-150 mg/dL
Glucose	118	70-120 mg/dL

**Figure 2:**
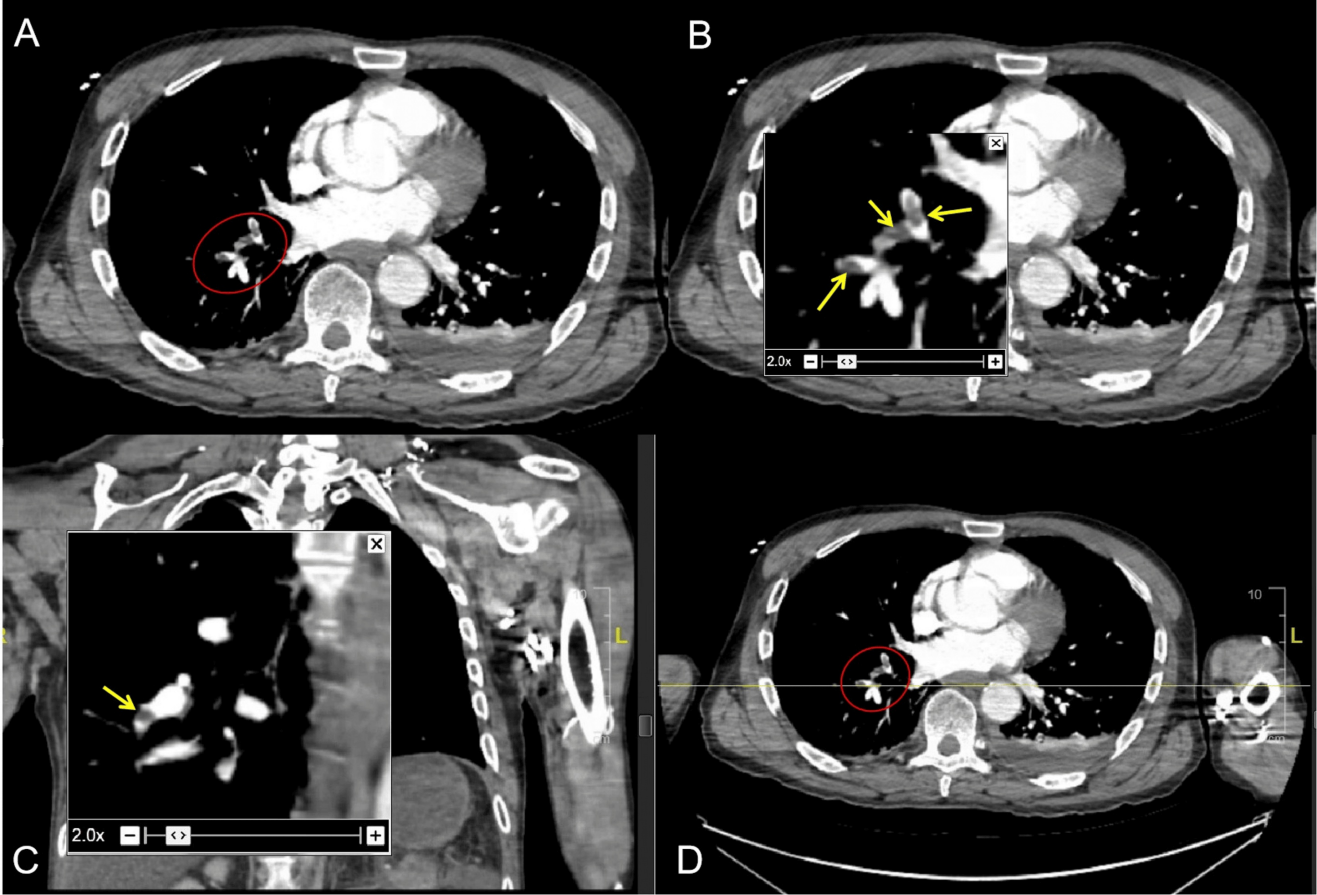
Computed pulmonary angiography Axial (A, B, and D) and coronal images (C) revealed emboli (red circles/yellow arrows) in segmental and subsegmental pulmonary arteries

**Figure 3:**
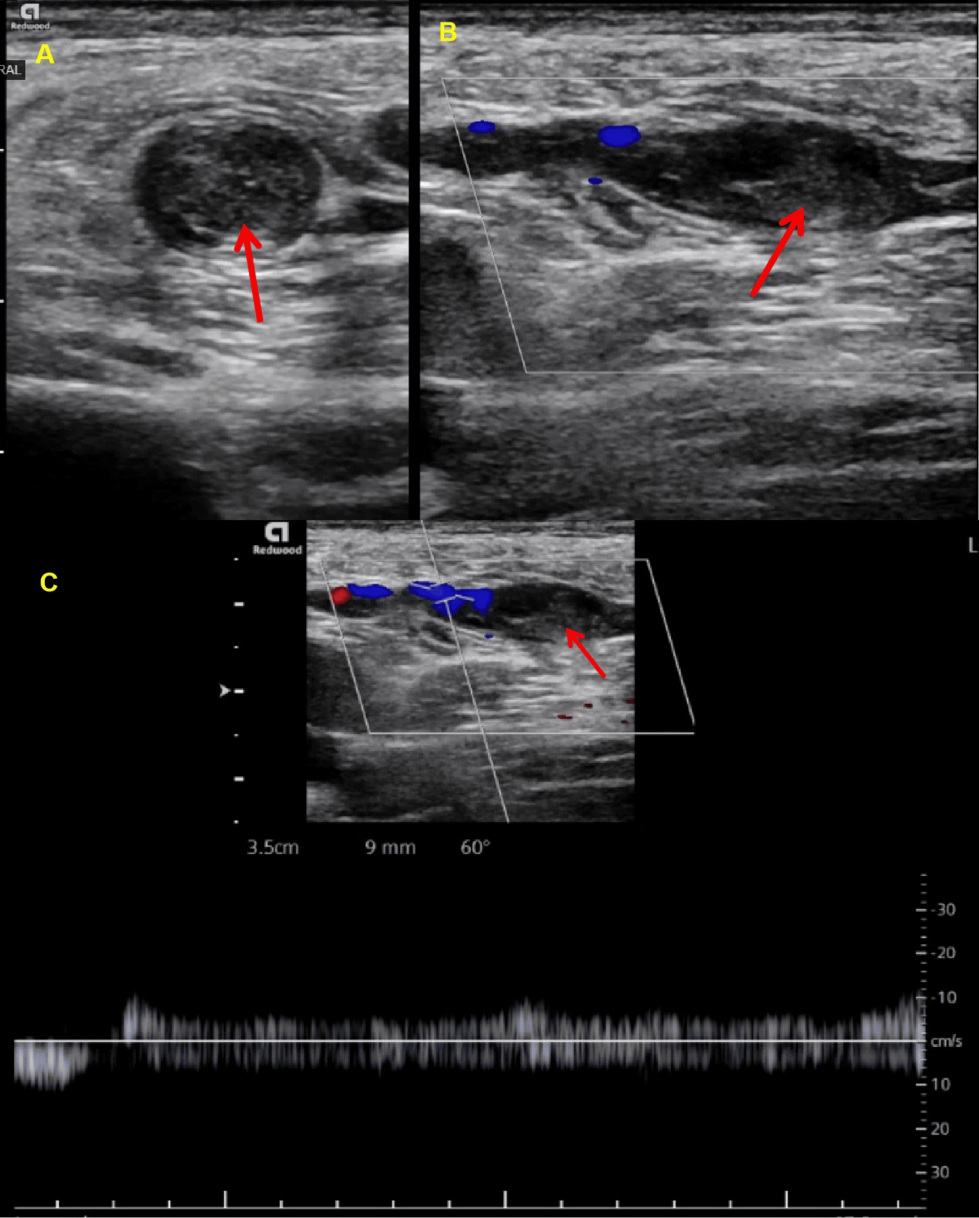
Left lower extremity ultrasound Cross-sectional (A) and longitudinal (B) images demonstrating luminal clots in the greater saphenous vein. (C) Demonstrates the flow void on color Doppler

At presentation, his Acute Physiology and Chronic Health Evaluation (APACHE) score was 12, Bedside Index for Severity in Acute Pancreatitis score was 2, and he had moderately severe acute pancreatitis based on the Revised Atlanta Classification. He was treated with intravenous hydration, thiamine supplements, benzodiazepines, antiemetics, and analgesics, with which he had symptomatic relief. However, tachycardia persisted, and he required nasal oxygen to maintain saturation, which prompted further evaluation. A CT pulmonary angiogram revealed scattered filling defects in the segmental and subsegmental pulmonary arteries of the right lower and left upper lobes, consistent with acute pulmonary emboli. There was no evidence of right heart strain; however, imaging revealed bilateral pleural effusions, with the left side more extensive than the right, bibasilar atelectasis, and emphysematous changes (Figure 2). At echocardiogram, the ejection fraction was normal with no demonstrable right heart strain or McConnell's sign. A subsequent venous ultrasound of the lower extremities identified thrombus formation in the left greater saphenous vein as well (Figure 3).

He was initiated on therapeutic dose enoxaparin for the extensive thrombotic venopathy. He improved with medical management and was subsequently discharged on vitamin supplements and apixaban. He remained asymptomatic at six weeks of follow-up, on a factor X inhibitor. Evaluation for hypercoagulable diathesis was performed on review and reported negative.

## Discussion

The pancreas, despite being a small intra-abdominal organ, is affected by diseases that have extensive ramifications, extending far beyond the abdominal cavity. Acute pancreatitis could impact virtually every organ system in the body, and the vascular system is no exception. A large retrospective study including patients over seven years reported a 26.5% incidence of vascular complications, with the most frequent being venous thrombosis of the splenic vein, portal vein, and superior mesenteric vein, in decreasing frequency. The study analyzed male gender, smoking, hyperlipidemia, high CTSI score, and nonvascular complications of pancreatitis as independent predictors for venous thrombosis. A median duration of 16 days was reported between the onset of pancreatitis and detection of venous thrombosis [5]. Spontaneous recanalization of thrombotic visceral veins was reported in 12.6% [5]. The overall incidence of pulmonary embolism and deep vein thrombosis complicating acute pancreatitis is 4% and 5%, respectively. Suryawanshi et al. reported a 3% incidence of combined pulmonary embolism and deep vein thrombosis in acute pancreatitis [6]. 

The mechanism of vascular complications in pancreatitis is complex. Conventionally, Virchow's triad describes the factors involved in thrombosis, which include hypercoagulability, endothelial dysfunction, and abnormalities in blood flow (stasis/turbulence) [7]. The pressure effect of inflamed tissue veins contributes to altered blood flow and an increased risk of thrombosis; however, this mechanism explains only regional thrombotic complications. On the contrary, endothelial injury related to the inflammatory response is believed to be the primary factor, which could explain the locoregional and distant thrombotic sequelae [8]. The role of hyperviscosity related to hemoconcentration in the pathogenesis is unclear. More severe pancreatitis, as evidenced by a higher APACHE and/or CTSI scores, and local or systemic complications of pancreatitis, correlates with a more severe inflammatory response and, expectantly, a higher risk of thrombotic complications; this explains the results of Guo et al. Study smoking is also associated with endothelial dysfunction and hence predisposes to thrombotic phenomena, applicable in patients with pancreatitis as well [9]. 

The management of splanchnic thrombotic complications in acute pancreatitis is unclear and lacks consensus [10]. Published data from some small-scale studies, however, do report higher rates of splanchnic venous recanalization in patients receiving therapeutic anticoagulants, without an increased risk of bleeding. In contrast, other studies have failed to replicate this benefit [5,10,11]. Zhang et al. evaluated catheter-based thrombolytic therapy versus systemic anticoagulation in a small cohort and reported higher recanalization rates and faster symptom resolution in patients with splanchnic vein thrombosis complicating acute pancreatitis. However, only six patients were included in the thrombolytic arm, limiting the adoption of this strategy without further evaluation [12]. 

Our patient with alcohol-induced pancreatitis and severe ethanol withdrawal was detected to have simultaneous distal superior mesenteric vein thrombosis, greater saphenous vein thrombosis, and pulmonary embolism, within a week of the onset of pancreatitis. The systemic inflammatory response and subsequent endothelial dysfunction should have elicited the thrombotic phenomenon, especially since the splenic and portal veins were spared. In this case, given pulmonary embolism, there was little doubt regarding the initiation of anticoagulation. Our patient was treated with low-molecular-weight heparin while in the hospital, which was later switched to apixaban at discharge. Additional evaluations failed to demonstrate any hypercoagulable state that could explain the thrombotic venopathy. We plan to follow up the patient on an outpatient basis, with imaging at three months and, being a provoked thrombosis, to hold off from anticoagulants once the thrombi are cleared or the veins are recanalized. This case is atypical, considering the early onset and multifocality of thrombosis, both of which are relatively uncommon in the setting of acute pancreatitis. 

## Conclusions

Venous thrombosis, especially involving splanchnic veins, is an underreported complication of acute pancreatitis, with independent predictors being male gender, smoking, and severity of pancreatitis. The inflammatory response, related to endothelial dysfunction, is believed to be the primary inciting factor for thrombotic sequelae in acute pancreatitis. The role of anticoagulation remains unclear in splanchnic venous thrombosis complicating acute pancreatitis. However, patients with extensive thrombosis beyond the visceral veins could benefit from anticoagulation. Availability of more data regarding thrombotic complications and their management is expected to guide experts to a consensus on the evaluation and management of these rare complications of acute pancreatitis.
